# Exploring the Association Between Glucose-6-Phosphate Dehydrogenase Deficiency and Autism Spectrum Disorder: A Narrative Review

**DOI:** 10.3390/children12081054

**Published:** 2025-08-11

**Authors:** Maitha Abdulla Alshamsi, Maitha Tareq Al Teneiji, Subhranshu Sekhar Kar, Rajani Dube

**Affiliations:** 1 RAK College of Medical Sciences, RAK Medical and Health Sciences University, Ras Al-Khaimah P.O. Box 11172, United Arab Emirates; 2 Department of Pediatrics, RAK College of Medical Sciences, RAK Medical and Health Sciences University, Ras Al-Khaimah P.O. Box 11172, United Arab Emirates; subhranshu.kar@rakmhsu.ac.ae; 3 Department of Obstetrics and Gynecology, RAK College of Medical Sciences, RAK Medical and Health Sciences University, Ras Al Khaimah P.O. Box 11172, United Arab Emirates; rajani.dube@rakmhsu.ac.ae

**Keywords:** G6PD deficiency, autism spectrum disorder, neurodevelopment, neuroinflammation, oxidative stress

## Abstract

Autism spectrum disorder (ASD) is a complex neurodevelopmental disease of multifactorial etiologies, manifesting as persistent challenges in social interactions, restrictive interests, and repetitive behaviors. Glucose-6-phosphate dehydrogenase (G6PD) deficiency is the most common human enzymopathy affecting red blood cell function. Although G6PD enzyme deficiency is known for its role in hemolytic anemia, emerging studies have suggested a potential association between G6PD deficiency and neurodegenerative and neurodevelopmental disorders, including autism. This narrative review explores the possible connection between G6PD deficiency and autism by analyzing relevant literature from the PubMed and Scopus databases. Current evidence points to plausible biological links, particularly oxidative stress and folate metabolism, warranting further investigation into G6PD deficiency as a potential risk modifier in ASD. Moreover, further research is necessary to elucidate the nature of this relationship and its implications for clinical practice.

## 1. Introduction

Autism Spectrum Disorder (ASD) is a neurodevelopmental condition characterized by persistent deficits in social communication and interaction, alongside restricted, repetitive patterns of behavior. The global prevalence has increased to approximately 1%, a trend attributed to expanded diagnostic criteria and heightened awareness [1,2]. ASD is diagnosed four times more often in males than in females, with underdiagnosis in females suspected due to subtler presentations [1,2,3]. It affects individuals across all racial and socioeconomic groups, though disparities in diagnosis persist [3,4,5]. The etiology of ASD is multifactorial, involving genetic, environmental, and neurological components. Around 15% of cases are linked to identifiable genetic mutations such as Fragile X syndrome, Rett syndrome, Down syndrome, and tuberous sclerosis [5,6,7]. Additional risk factors include prenatal and perinatal influences like infections, exposure to valproate, advanced paternal age, low birth weight, and obstetric complications [7]. Pathophysiologically, the neural mechanisms underlying the impairments observed in ASD remain unknown. ASD is highly heritable [8]. Mutations in genes such as SHANK3, NRXN1, NLGN3, and MECP2 are critical for synaptic function which are commonly implicated [8,9]. Neuroimaging studies reveal early-life macrocephaly and abnormalities in cerebral cortical organization and connectivity, with local hyperconnectivity and long-range hypoconnectivity [10]. Neurochemical imbalances, especially involving GABA, glutamate, and serotonin, as well as evidence of neuroinflammation and microglial activation, also contribute to ASD’s pathogenesis. Environmental toxins (e.g., heavy metals, pesticides), prenatal infections, and maternal drug exposure have been investigated for their contributory roles. Epigenetic modifications, including altered DNA methylation and histone modification, may further influence gene expression associated with ASD [6,7,8,11,12]. Clinically, early signs may appear in infancy, such as limited eye contact, minimal babbling, and lack of social interest. By age two, affected children may exhibit repetitive behaviors, language regression, difficulty with peer interaction, and rigid routines [7,8,11,12]. They often display impairments in social reciprocity, communication challenges including pedantic speech, lack of gesturing, and abnormal body language. Neurological manifestations include seizures, motor tics, and gaze abnormalities, while physiological symptoms may encompass altered sensory processing, reduced pain sensitivity, and abnormal thermoregulation [5]. Behavioral issues such as irritability, tantrums, hyperactivity, and aggression are common, though some individuals may demonstrate exceptional talents in memory or mathematics [4,5]. Management of ASD is multidisciplinary and individualized, aiming to enhance function and reduce symptoms, especially through early intervention during preschool years [12,13]. Key strategies include behavioral therapies like Applied Behavior Analysis (ABA), structured educational programs, and family-centered support to foster communication and social skills [14]. Speech, occupational, and physical therapies are integral, while pharmacological agents may be used to manage severe behaviors or comorbidities like anxiety or hyperactivity [13,14]. Comorbid conditions such as epilepsy, gastrointestinal disturbances, and sleep disorders should also be addressed [15]. Family involvement is essential, and psychoeducation helps caregivers manage daily challenges and promote development [16].

Glucose-6-Phosphate Dehydrogenase (G6PD) deficiency is the most prevalent hereditary enzymopathy of red blood cells, affecting approximately 400 million people worldwide [17]. It is a genetic disorder inherited in an X-linked recessive mode, predominantly affecting males. Females may exhibit symptoms if they are homozygous for the mutated gene (having two copies), or if they are heterozygous (having one copy) with inactivation of the normal X chromosome [18]. The World Health Organization (WHO) categorizes the G6PD enzyme into five classes based on level of enzyme activity and the severity of clinical symptoms. Class I is the most severe, with less than 10% of normal enzyme activity, leading to chronic non-spherocytic hemolytic anemia. Class II is the Mediterranean variant, with less than 10% enzyme activity, but causes intermittent hemolysis. Class III represents a moderate deficiency with enzyme activity between 10% and 60%. Classes IV and V are asymptomatic, characterized by normal and increased enzyme activity, respectively [19]. G6PD enzyme plays an important role in the transformation of oxidized nicotinamide adenine dinucleotide phosphate (NADP) into its reduced form, nicotinamide adenine dinucleotide phosphate hydrogen (NADPH). The production of NADPH is essential for maintaining the reduced form of glutathione (GSH). GSH is a cellular antioxidant that facilitates the conversion of harmful oxygen-free radicals into harmless substances [20]. In G6PD-deficient individuals, this biochemical process is disrupted, leading to insufficient levels of NADPH and consequently inadequate levels of glutathione. The lack of GSH results in the accumulation of reactive oxygen species during exposure to oxidative stressors [21,22]. A wide clinical spectrum associated with G6PD deficiency has been identified. G6PD deficiency is the most common cause of neonatal jaundice [23]. G6PD-deficient newborns present with persistent neonatal jaundice, which, if left untreated, remains a leading cause of kernicterus or bilirubin-induced neurological dysfunction (BIND) [24]. Additionally, exposure to oxidative stressors may result in an acute hemolytic reaction. These stressors include certain medications, consumption of foods like fava beans, exposure to specific chemicals, or infections [25]. Accelerated hemolysis leads to sudden decrease in RBC count, resulting in symptoms such as fatigue, pallor, jaundice, dark urine, and in severe cases, shortness of breath and an enlarged spleen [26]. The severity of hemolytic anemia varies significantly among G6PD-deficient individuals, depending on their sensitivity to oxidants, which is in turn caused by variations in G6PD enzyme level. A chronic hemolytic anemia (CNSHA) in G6PD deficiency usually results from severe enzymatic deficiency associated with WHO class I. Unlike acute hemolytic episodes triggered by infections, foods, or medications, CNSHA presents continuously and necessitates ongoing management to prevent complications [27]. Management of G6PD deficiency necessitates a comprehensive approach aimed at preventing hemolytic episodes and addressing complications immediately. Avoidance of known triggers that can induce hemolysis is considered the cornerstone of treatment [26]. Neonates developing significant jaundice require phototherapy or exchange transfusion to reduce bilirubin levels and prevent kernicterus. G6PD-deficient individuals developing mild hemolysis are managed conservatively with supportive therapy. However, severe cases may require blood transfusions [24].

Recent discoveries have highlighted the importance of G6PD enzyme in neuroprotection, beyond its established role in maintaining proper function in red blood cells. This review aims to critically evaluate the existing evidence for an association between G6PD deficiency and ASD, focusing on proposed pathophysiological mechanisms including oxidative stress, impaired folate metabolism, and neuroinflammation. Together, these mechanisms offer insights into the potential connections between G6PD deficiency and ASD.

## 2. Materials and Methods

### 2.1. Objective

This review investigates the association between G6PD deficiency and ASD by synthesizing existing literature. The review includes studies that explore the clinical correlates of G6PD deficiency in relation to autism.

### 2.2. Inclusion Criteria

#### 2.2.1. Study Type

Peer-reviewed articles, case studies, and clinical reports that discuss G6PD deficiency and its potential link to ASD.

#### 2.2.2. Population

Studies involving children diagnosed with ASD and those with confirmed G6PD deficiency.

#### 2.2.3. Geographic Scope

No restriction; studies from all regions were eligible, in order to capture diverse perspectives.

#### 2.2.4. Language

Studies and reviews published in English that examined the relationship between G6PD deficiency and ASD.

#### 2.2.5. Time Frame

Studies conducted from inception until February 2025.

### 2.3. Exclusion Criteria

#### 2.3.1. Non-Peer-Reviewed Sources

Articles lacking rigorous scientific validation, opinions and editorials.

#### 2.3.2. Duplicate Studies

Multiple publications from the same dataset without new or additional findings.

#### 2.3.3. Animal or In Vitro Studies

Studies conducted using non-human subjects, and laboratory-based research.

### 2.4. Literature Search Strategy

A comprehensive literature search was conducted across two databases, PubMed and Scopus. The search incorporated MeSH terms and relevant keywords, including the following: “G6PD deficiency,” “autism,” and “neurodevelopmental disorders.” The search employed Boolean operator (AND) to ensure comprehensive coverage.

Two reviewers independently screened the search results for relevance. They assessed the titles and abstracts, followed by a full-text evaluation of potentially eligible articles. Discrepancies were resolved through discussion or consultation with the third reviewer.

**Figure 1:**
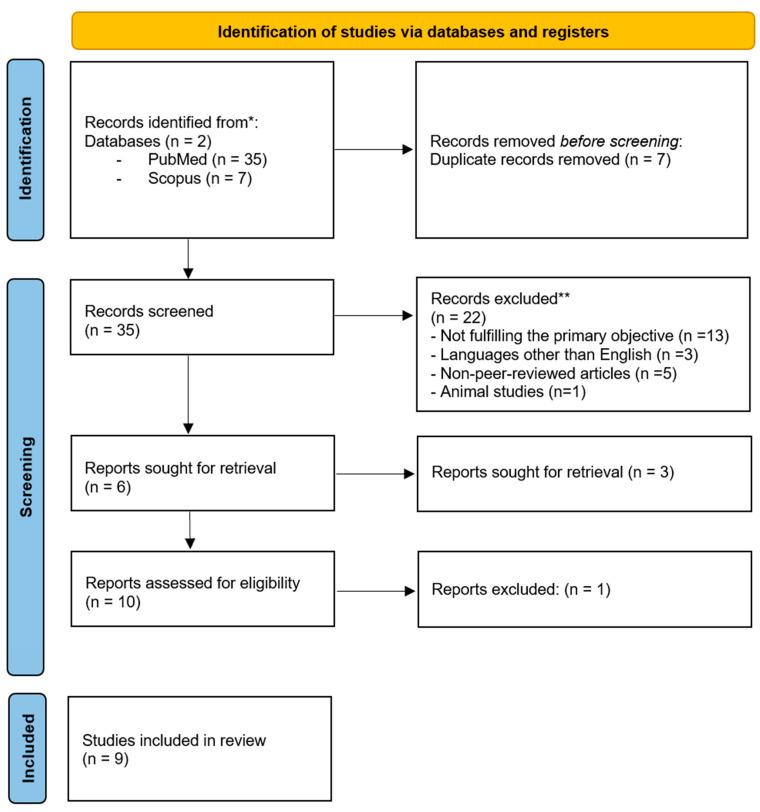
PRISMA flow diagram for selection of studies. * Two electronic databases were searched. The number of records identified from each database is reported separately. ** No automation tools were used for screening or exclusion; all records were assessed manually by reviewers.

The PRISMA flow diagram illustrates the study selection process (Figure 1). A total of 42 records were initially retrieved (35 articles from PubMed and 7 articles from Scopus), out of which 7 were excluded due to duplication, leaving 35 articles for screening. Based on title and abstract screening, 22 studies were excluded, leaving 15 articles. Of these, 6 were sought for retrieval, but 3 full-text articles could not be retrieved. The remaining 10 full-text articles were assessed for eligibility. In total, 1 out of 10 articles did not meet the inclusion criteria and was excluded from the review.

### 2.5. Data Extraction and Analysis

Data were extracted from selected studies, including Author(s) and publication year, study type and design, population characteristics, key findings related to G6PD deficiency and ASD.

## 3. Results

A total of nine studies were included in Table 1, exploring the relationship between G6PD deficiency and ASD, as well as broader cognitive and neurodevelopmental outcomes. Mondal et al. (2021) [28] suggested that reduced NADPH levels in individuals with G6PD deficiency may contribute to oxidative stress implicated in ASD pathogenesis. Similarly, Al-Salehi et al. (2009) [29] reported a potential association between G6PD and the core triad of autism symptoms.

Other studies focused on the indirect effects of G6PD deficiency, particularly through the risk of neonatal hyperbilirubinemia, which is associated with long-term neurodevelopmental impairments, including autism, intellectual disability, and sensorineural hearing loss (Olusanya et al., 2014 [30]; Lin et al., 2022 [31]; Olusanya et al., 2015 [32]).

Case reports and observational studies further supported a possible connection between G6PD deficiency and broader neurocognitive challenges. For instance, Alagoz et al. (2020) [33] described a child with G6PD deficiency and severe neurological manifestations including developmental regression and learning difficulties.

Studies by Dowd (1980) [34], Toren et al. (1994) [35], and Seth et al. (1981) [36] observed a higher prevalence of G6PD deficiency among individuals with intellectual disability or mental retardation, suggesting a potential link between G6PD deficiency and limited cognitive function. These findings collectively point toward a possible role of G6PD deficiency in neurodevelopmental vulnerability, though further focused research is needed to establish causality.

## 4. Discussion

G6PD deficiency is an X-linked recessive disorder, meaning the gene responsible for the enzyme deficiency is located on the X chromosome. As males have only one X chromosome, they are more likely to express this deficiency if they inherit the affected gene from their mothers because they lack a second X chromosome to potentially counteract the defect. This genetic trait explains why G6PD deficiency is significantly more common in males than in females [37]. Similarly, ASD also exhibits a higher prevalence in males. The male-to-female ratio in autism is estimated to be about 4:1, suggesting that males are four times more likely to be diagnosed with ASD than females [38]. While the exact reasons for this gender disparity remain under investigation, several hypotheses have been proposed. Some theories suggest that genetic, hormonal, or environmental factors may play a role with males potentially being more vulnerable to certain neurodevelopmental challenges [39]. The shared gender predilection in both G6PD deficiency and ASD raises intriguing questions about the possible genetic or biochemical mechanisms that could link these conditions, particularly in males, where the interplay between genetic susceptibility and environmental triggers may be more pronounced. Supporting this hypothesis is a case series from Saudi Arabia reporting two individuals with G6PD deficiency and autism [29].

**Figure 2:**
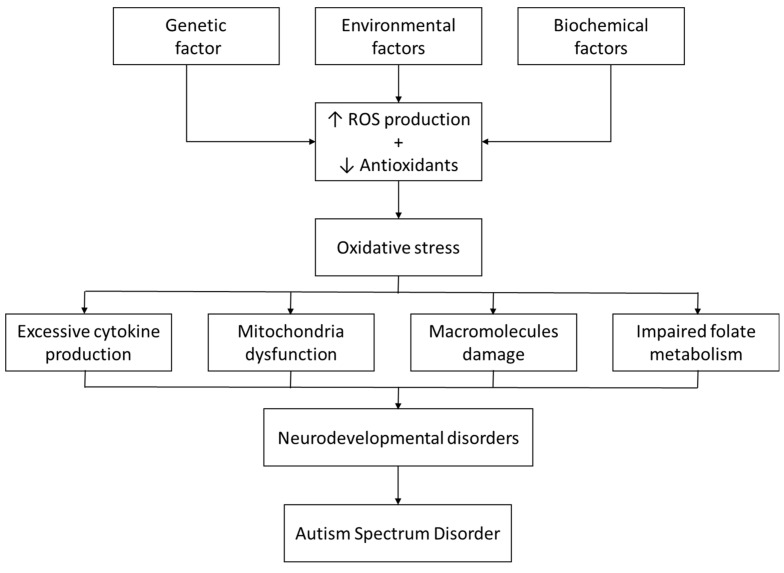
Proposed Mechanisms of Oxidative Stress in Autism Spectrum Disorder. ↑ Increase, ↓ Decrease.

Additionally, G6PD enzyme deficiency is implicated in various physiological and biochemical disruptions including impaired folate metabolism, disturbed oxidative equilibrium, and neuroinflammation. These alterations may be directly or indirectly related to the pathogenesis of ASD (Figure 2) [40].

### 4.1. Oxidative Disequilibrium

Reactive oxygen species (ROS) are continuously produced in aerobic organisms as by-products of normal oxygen metabolism. Mitochondria are the main source of endogenous ROS production. Exogenous factors that contribute to ROS production include ionizing radiation, ultraviolet light, and various oxidizing chemicals. At optimal concentrations, ROS are crucial for various physiological processes. Phagocytosis process by immune cells such as neutrophils, macrophages, and monocytes, releases free radicals to destroy invading pathogens as part of the immune response. ROS also serve as essential second messengers in cell signaling processes. However, elevated ROS concentrations for a prolonged time can damage cellular macromolecules such as DNA, proteins, lipids, and lipoproteins leading to necrosis and apoptosis. It may also cause mitochondrial dysfunction.

Oxidative stress arises from an imbalance between ROS production and the cell’s antioxidant defenses, either due to increased ROS generation or compromised antioxidant systems. This imbalance, along with the resulting biochemical alterations in macromolecules and dysfunctional mitochondria, are implicated in various pathological conditions and human diseases [41,42]. The developing brain is particularly more susceptible to oxidative stress, which is the cornerstone event in all pathways implicated in the development of neurodevelopmental disorders [43]. Markers of oxidative stress such as lipid peroxide, malondialdehyde (MDA), protein carbonyl, and 3-nitrotyrosine (3-NT), 8-hydroxydeoxyguanosine (8-OHdG) are elevated in children with ASD [44,45]. However, low levels of antioxidant proteins such as transferrin and ceruloplasmin have been reported in children with ASD [46].

### 4.2. Impaired Folate Metabolism

Folate metabolism plays a critical role in maintaining cellular functions, particularly in the synthesis and repair of DNA [46]. In G6PD-deficient individuals, the reduced availability of NADPH leads to an increased vulnerability to oxidative damage, as the cells have a diminished capacity to neutralize ROS. This oxidative stress can impair folate metabolism, which relies on NADPH for the regeneration of tetrahydrofolate (THF). THF is an active form of folate crucial for nucleotide biosynthesis and methylation reactions [47]. Furthermore, folate is vital for the synthesis of neurotransmitters and the maintenance of myelin, the protective sheath around neurons. Also, disruption in folate metabolism can lead to elevated homocysteine levels, a neurotoxic amino acid linked to cognitive decline, neurodegenerative diseases, and psychiatric traits [48,49]. A metanalysis indicated that maternal use of folic acid supplements during pregnancy could substantially lower the risk of ASD in offspring, compared to women who did not take folic acid supplements [50]. Another study showed evidence of improved verbal communication with a 12-week high-dose folinic acid compared with placebo [51].

Folate receptor α (FRα) is the primary transporter for folate across the blood brain barrier. The activity of this transporter may be impaired in patients with ASD resulting in lower concentrations of folate in the cerebrospinal fluid (CSF) compared to the blood. This discrepancy is known as cerebral folate deficiency (CFD). The commonest two mechanisms behind FRα dysfunction are autoantibody formation and mitochondrial diseases [52,53,54].

### 4.3. Neuroinflammation

Cytokines are chemical mediators produced by cells upon exposure to antigens or immunogens. These fast-acting signaling molecules are crucial for regulating the balance between pro-inflammatory and anti-inflammatory responses. Cytokines and their receptors play key roles in the development of the central nervous system (CNS). They influence processes such as synapse formation, maturation, and elimination, as well as cell renewal, survival, and apoptosis in CNS cells, including neurons, microglia, astrocytes, and oligodendrocytes. Disruption in cytokine expression during critical developmental periods can have a profound effect on the CNS. Elevated levels of inflammatory cytokines have been frequently linked to ASD [55].

Maternal immune activation (MIA)—in which cytokines that are produced in the maternal serum in response to infections or immune system dysregulation are transferred to the fetal brain across the placenta—is further evidence that supports this hypothesis. MIA disrupts normal neurodevelopmental processes such as synaptogenesis and neuronal differentiation, resulting in wide range of neuropsychiatric disorders in the offspring such as ASD, intellectual disability (ID), major depressive disorder (MDD), bipolar disorder, and schizophrenia [45].

## 5. Conclusions

In conclusion, our review highlights the link between G6PD deficiency and ASD, reflecting a complex interaction of genetic, biochemical, and environmental elements. Both G6PD deficiency and ASD share a gender predilection in which they are more commonly seen in males. Oxidative stress, a known consequence of G6PD deficiency, is seen in individuals with ASD, where it contributes to cellular damage and mitochondrial dysfunction. Furthermore, impaired folate metabolism, which is essential for DNA synthesis, repair, and neurotransmitter production, may further exacerbate neurodevelopmental abnormalities in G6PD-deficient individuals, potentially increasing the likelihood of ASD. Lastly, chronic neuroinflammation, driven by immune dysregulation, has been implicated in both conditions and may disrupt critical neurodevelopmental processes. This review suggests potential mechanistic links between G6PD deficiency and ASD, particularly through shared pathways involving oxidative stress, folate metabolism, and immune dysfunction. Further research is needed to clarify the exact mechanisms and to explore potential therapeutic approaches.

## Figures and Tables

**Table 1:** Data extraction. CIC: Capicua transcriptional repressor.

Author and Publication Year	Study Design	Population Characteristics	Key Findings
Mondal et al., 2021 [28]	Literature review	Not mentioned	ASD is associated with a low level of NADPH, which suggests that the deficiency of G6PD is one of the major contributing factors for dysregulation of oxidative balance in pathogenesis of ASD.
Olusanya et al., 2014 [30]	Literature review	Not mentioned	Severe neonatal hyperbilirubinemia is frequently associated with neonatal mortality and neurodevelopmental disorders in countries with significant G6PD deficiency.
Lin et al., (2022) [31]	Systematic review and meta-analysis	Not mentioned	G6PD deficiency was identified as a risk factor for neonatal hyperbilirubinemia (OR = 1.62, 95% CI: 1.44, 1.81, Z = 8.39, *p* < 0.00001). Hyperbilirubinemia may lead to severe complications, including lifelong disability such as growth retardation, encephalopathy, autism and hearing impairment.
Al-Salehi et al., 2009 [29]	Case series	49 subjects (37 males & 12 females) have a typical triad of autism symptoms; social deficits, communication impairment & rigid ritualistic interest.	There is a possible correlation between G6PD deficiency and autism. Potentially through bilirubin induced brain damage causing neuropathological changes.
Olusanya et al., 2015 [32]	Systematic Review and Meta-Analysis	Not mentioned	Infants with G6PD deficiency have an elevated risk of severe hyperbilirubinemia (OR, 8.01; 95% CI, 2.09–30.69, *p* = 0.002). Surviving infants may acquire long-term neurodevelopmental sequelae such as cerebral palsy, sensorineural hearing loss, intellectual difficulties or gross developmental delays
Alagoz et al., 2020 [33]	Case report	8-year-old boy with hemizygous variation in G6PD gene and heterozygous mutation in CIC gene	The child suffers from focal epileptiform activity and hypsarrhythmia in electroencephalography (EEG), seizures, psychomotor retardation, speech impairment, intellectual disability, developmental regression, and learning difficulties.
B Dowd et al., 1980 [34]	Observational, screening-based study on a clinical population.	100 patients who were classified as mentally retarded and who were hospitalized under state care.	The screening and subsequent statistical analysis of the data indicates that the incidence of G-6-PD is drastically higher among Caucasian males in the atypical population than is to be expected and it is somewhat higher among the atypical Negroes. indicates strongly that there may be a relationship between G-6-PD deficiency and limited mental capacity.
Toren et al., 1994 [35]	Case series	6 children of one family who are deficient of adenylate kinase enzyme and in three of them a combined G6PD deficiency was found.	Five of these patients suffer from mild mental retardation and the sixth is severely retarded. The mildly retarded patients have limited learning abilities, poor performance at school and a limited vocabulary.
P K Seth, et al. 1981 [36]	Observational study	152 mentally retarded individuals and control group 156	Of the mentally retarded individuals, 11.18% were found to be deficient in glucose-6-phosphate dehydro-genase compared with 2.56% of the controls. This indicates a possible association between G6PD deficiency and mental subnormality.
